# A Note on Vestigial Osmotic Pressure

**DOI:** 10.3390/membranes13030332

**Published:** 2023-03-14

**Authors:** Hao Wu, Zhong-Can Ou-Yang, Rudolf Podgornik

**Affiliations:** 1 Wenzhou Institute, University of Chinese Academy of Sciences, Wenzhou 325001, China; 2 Institute of Theoretical Physics, Chinese Academy of Sciences, Beijing 100190, China; 3 School of Physical Sciences, University of Chinese Academy of Sciences, Beijing 100049, China; 4 Kavli Institute for Theoretical Sciences, University of Chinese Academy of Sciences, Beijing 100049, China; 5 Institute of Physics, Chinese Academy of Sciences, Beijing 100190, China

**Keywords:** Helfrich free energy, topological changes, spontaneous tension, osmotic pressure

## Abstract

Recent experiments have indicated that at least a part of the osmotic pressure across the giant unilamellar vesicle (GUV) membrane was balanced by the rapid formation of the monodisperse daughter vesicles inside the GUVs through an endocytosis-like process. Therefore, we investigated a possible osmotic role played by these daughter vesicles for the maintenance of osmotic regulation in the GUVs and, by extension, in living cells. We highlighted a mechanism whereby the daughter vesicles acted as osmotically active solutes (osmoticants), contributing an extra vestigial osmotic pressure component across the membrane of the parent vesicle, and we showed that the consequences were consistent with experimental observations. Our results highlight the significance of osmotic regulation in cellular processes, such as fission/fusion, endocytosis, and exocytosis.

## 1. Introduction

In osmotic-stress experiments on lipid multilayers [1], where the osmotic pressure was regulated by dissolved PEG osmoticant and the inter-bilayer separation was measured by small-angle X-ray scattering (SAXS) [2], it was found that, in general, the samples exhibited smaller bilayer separations in a completely unstressed state, which was defined by the vanishing of the osmotic pressure P=0, than at the smallest finite osmotic stresses applied by the PEG osmoticant. It was proposed that unilamellar vesicles could peel off during sample preparation and could constitute another osmotically active component of the solution. The osmotic pressure exhibited by the peeled-off vesicles was dubbed the vestigial osmotic pressure and added to the osmotically active dissolved PEG. The total osmotic pressure would then be governed by the sum of the direct PEG-related osmotic pressure, under full experimental controls, and the vestigial osmotic pressure, which was contingent upon the method of sample preparation.

In a recent experiment, DOPC giant unilamellar vesicles (GUVs) were prepared in pure water without any buffer or solutes, which allowed for in situ observations directly after changing the bathing solution and adding glucose to the solution [3]. In this case, the sugar molecules could induce spontaneous curvatures, and the GUVs rapidly deformed to a prolate shape at an osmotic pressure of up to 0.3 atm while remaining stable for several hours under these solution conditions. At even higher osmotic pressures, from 0.2 atm to 0.4 atm, subsequent rinsing resulted in the formation of internal “daughter” vesicles, which were fully disconnected from the parent lipid bilayer, with an interior solution originating from the bulk liquid and having an approximate radii of a factor of 5 smaller than the original GUVs. At a lower osmotic pressure of 0.1 atm, no such changes were discernible. While similar results had been reported previously [4,5], the observation of the disconnected progeny inside the parent vesicle was unique.

In the following article, we describe a simple model for the osmotic action of the “daughter” vesicles inside giant DOPC unilamellar vesicles (GUVs). The daughter vesicles were assumed to be completely disconnected from each other as well as from the parent vesicle and, thus, represented a different topological state. The model was based on the curvature-free energy that also included a Gaussian curvature term because the formation process of the daughter vesicles involved topological changes. The osmotic actions of the daughter vesicles were approximated at the lowest order by the simplest Van’t Hoff osmotic pressure. Though more detailed calculations could be performed, we showed that even at the simplest level, the results yielded reasonable values for relevant observations.

## 2. Osmotic Pressure Equilibrium

**Figure 1:**
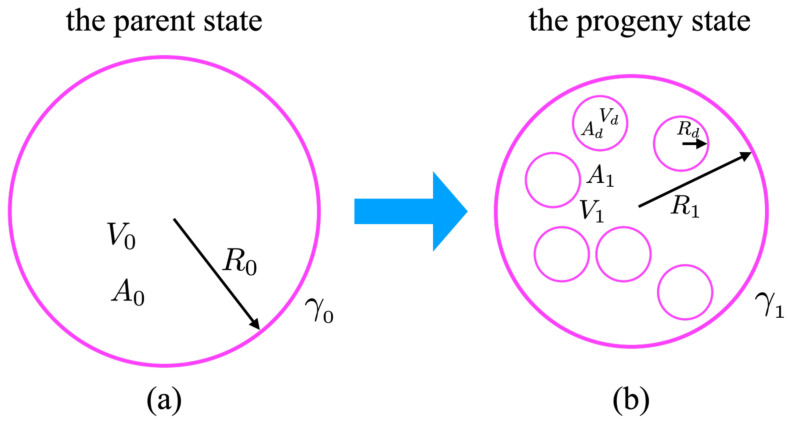
(**a**) A schematic depiction of the “parent”, a single spherical vesicle with the fixed radius $R_{0}$, area $A_{0}$, and volume $V_{0}$ (the parent state), converting to (**b**) the “progeny” spherical vesicle with the fixed radius $R_{1}$, area $A_{1}$, and volume $V_{1}$, including *n* daughter spherical vesicles with the fixed radius $R_{d}$, area $A_{d}$, and volume $V_{d}$ (the progeny state).

In our model, we assumed and analyzed two states of a single-component lipid bilayer GUV and disregarded the effect of thermal fluctuations [6]. The parent state (designated as “0”, as shown in Figure 1a) was devoid of any spontaneous curvature and its curvature-free energy was described by its Helfrich ansatz in the form:

(1) $$\begin{array}{c} F_{0}=\frac{1}{2}K_{C}\oint_{A_{0}}{\left(2H\right)}^{2}\;dA+K_{G}\oint_{A_{0}}K\;dA+\gamma_{0}\oint_{A_{0}}\;dA+P_{0}\int_{V_{0}}dV, \end{array}$$

where $K_{C}$ is the bending modulus. We specifically included the Gaussian curvature term with the corresponding modulus, $K_{G}$, as the emergence of the daughter vesicles involved a topological change, to which this term was sensitive.

Two Lagrangian mutipliers, $\gamma_{0}$ and $P_{0}$, were chosen to fix the area $A_{0}$ and the volume $V_{0}$ of the vesicle [7]. For the spherical shape characterized by the radius *R*, we used the simplified forms of the mean curvature and the Gaussian curvature as:

(2) $$\begin{array}{c} H=\frac{1}{2}\left(C_{1}+C_{2}\right)=\frac{1}{R}\;\mathrm{and}\;K=C_{1}C_{2}=\frac{1}{R^{2}}.\;\; \end{array}$$

The shape equation was obtained from the first variation of the Helfrich free energy and was determined as the solution of the Euler-Lagrange equation:

(3) $$\begin{array}{c} P_{0}-2\gamma_{0}H+2K_{C}H\left(2H^{2}-2K\right)+2K_{c}\Delta_{s}H=0, \end{array}$$

where $\Delta_{s}$ is the surface Laplace-Beltrami operator [8,9,10]. After delimiting the spherical vesicle shapes, the terms involving the surface Laplacian vanished.

In our model, the progeny state with internal daughter vesicles (designated as “1”, as shown in Figure 1b) described the system after the introduction of glucose molecules into the bathing solution. We presumed that the only effect of adding the glucose molecules was to induce spontaneous curvature by interacting with the lipid bilayer [7,11]. This was also confirmed at lower osmotic pressures when the addition of sugar induced a prolate shape, while at higher osmotic pressure, it resulted in the formation of internal daughter vesicles [3]. Therefore, we assumed that the progeny state would be characterized by a finite, if small, spontaneous curvature. In this state, the Helfrich free energy could be written in the form:

(4) $$\begin{array}{c} F_{1}=\frac{1}{2}K_{C}\oint_{A_{0}}{\left(2H+C_{0}\right)}^{2}\;dA+K_{G}\oint_{A_{0}}K\;dA+\gamma_{1}\oint_{A_{0}}\;dA+P_{1}\int_{V_{1}}dV, \end{array}$$

since the total area was conserved, i.e., $A_{0}=A_{1}$, but the volume was not. The sign of $C_{0}$ was consistent with the experiment where the spontaneous curvature appeared to be negative.

The Euler-Lagrange equation for the shape then was expressed, as follows:

(5) $$\begin{array}{c} P_{1}-2\gamma_{1}H+K_{C}\left(2H+C_{0}\right)\left(2H^{2}-C_{0}H-2K\right)+2K_{C}\Delta_{s}H=0. \end{array}$$

Once again, the last term vanished if we assumed that the state ”1“ consisted exclusively of spherical vesicle shapes, whether they were the parent or the daughter vesicles. The conservation of the total area in the system undergoing the change from State 0→ State 1 could then be written as:

(6) $$\begin{array}{c} A_{0}=A_{1}+nA_{d}\;\mathrm{or}\;R_{0}^{2}=R_{1}^{2}+nR_{d}^{2}, \end{array}$$

where *n* is the number of disconnected daughter vesicles in the formation state and $R_{d}$ is their radius, which was assumed to be the same for all.

The Helfrich free energies for the parent and progeny states of spherical vesicles with constant curvatures could then be written as follows. In the initial state of a spherical parent vesicle, the elastic free energy is:

(7) $$\begin{array}{c} F_{0}=8\pi K_{C}+4\pi K_{G}+\gamma_{0}A_{0}+P_{0}V_{0} \end{array}$$

While in the final state, the interior of the shrunken parent vesicle contained a progeny of small, spherical daughter vesicles, well separated from one another. The elastic energy in this scenario was expressed by the following:

(8) $$\begin{array}{c} F_{1}=8\pi K_{C}{\left(1+C_{0}R_{1}/2\right)}^{2}+8\pi nK_{C}{\left(1+C_{0}R_{d}/2\right)}^{2}+4\pi \left(n+1\right)K_{G}+\gamma_{1}A_{0}+P_{1}V_{1}. \end{array}$$

Based on the above, we obtained $A_{0}=4\pi R_{0}^{2}$, $V_{0}=\frac{4}{3}\pi R_{0}^{3}$ and $V_{1}=\frac{4}{3}\pi R_{1}^{3}$. The spontaneous curvature $C_{0}$ originated from the interaction of the glucose molecules with the bilayer. We expressed the differences in the Helfrich energy between the parent and progeny states as:

(9) $$\begin{array}{ccc} \Delta F=F_{1}-F_{0} & = & 8\pi nK_{C}+8\pi K_{C}C_{0}\left(R_{1}+nR_{d}\right)+2\pi K_{C}C_{0}^{2}R_{0}^{2}\\ & & +\;4\pi nK_{G}+\gamma_{\mathrm{sp}}A_{0}+P_{1}V_{1}-P_{0}V_{0}, \end{array}$$

where the difference $\gamma_{1}-\gamma_{0}$ is the glucose-induced spontaneous tension [12].

(10) $$\begin{array}{c} \gamma_{1}-\gamma_{0}=\gamma_{\mathrm{sp}}. \end{array}$$

The expression $\gamma_{\mathrm{sp}}=K_{C}C_{0}^{2}/2$ was recently reported in [12], in which the spontaneous curvature was one half of $C_{0}$ in their work due to different free-energy coefficients. However, the above ansatz did not contain any details of the energies involved in the vesicle formation, apart from the curvature energy.

In general, the difference in free energies can be of either sign. However, in a thermal equilibrium, a spontaneous generation of a progeny state with n≠0 could only occur if the difference in the free energies was negative, i.e., ΔF≤0, with the critical isotherm corresponding to ΔF=0. Next, we analyzed this critical isotherm.

The connection between the two osmotic pressures of the parent and progeny states, $P_{0}$ and $P_{1}$, respectively, and the radii of the vesicles in the two states was obtained according to Euler-Lagrange equations. Furthermore, it was straightforward to deduce from the shape calculation in of Equation (3) that:

(11) $$\begin{array}{c} P_{0}-\frac{2\gamma_{0}}{R_{0}}=0, \end{array}$$

In addition, based on the shape calculation in Equation (5), we could assume conversely that:

(12) $$\begin{array}{c} P_{1}-\frac{2\gamma_{1}}{R_{1}}-\frac{K_{C}C_{0}^{2}}{R_{1}}+\frac{2K_{c}C_{0}}{R_{1}^{2}}=0. \end{array}$$

According to these definitions, we associated the differences between the osmotic pressures $P_{0}$ and $P_{1}$ with the vestigial osmotic pressure differences. We assumed that it was a consequence of the osmotic action of the freely mobile *n* daughter vesicles in the progeny state, and we disregarded any other contributions to the osmotic pressure. Formally, the vestigial osmotic pressure differences of the *n* daughter vesicles could be written as:

(13) $$\begin{array}{c} \delta P=P_{1}-P_{0}=\frac{n}{V_{1}}\left(k_{B}T\right)\phi \left(\frac{n}{V_{1}}\right) \end{array}$$

where $\phi \left(\frac{n}{V_{1}}\right)$ is the osmotic coefficient as a function of the density of the daughter vesicles, and $\frac{n}{V_{1}}\left(k_{B}T\right)$ is the Van’t Hoff ideal osmotic pressure. In a recent experimental work, the daughter vesicles were well separated in the parent vesicle [3], and we assumed that the interactions between them were negligible. Next, we set $\phi \left(\frac{n}{V_{1}}\right)=1$; however, more sophisticated models have considered that this scenario could be classified as a small system [13], and these could be used to account for various effects that we disregarded in this study.

## 3. Results and Discussion

We solved the above equations for the critical isotherm, defined specifically as ΔF=0 for $R_{1}$ and *n* as functions of the original radius $R_{0}$, all based on experiments. The stable transition occurred for ΔF≤0, and the critical isotherm delimited the region where the progeny formed inside the parent vesicle.

Since we wanted to compare the most recent experiments on DOPC GUVs [3], all the numerical values were used as the DOPC bilayer measurements, namely as $K_{c}=20k_{B}T$ [14,15,16], $K_{G}=-15k_{B}T$ [15,16], $\gamma_{0}=0.01\;\mathrm{mN}/m$ [17], $\gamma_{sp}=7.05\times 10^{-6}\;\mathrm{mN}/m$, which corresponded to $C_{0}=0.206$ μm ${}^{-1}$ falling within the range in [7], T=300 K (room temperature). Based on the experiment in [3], the statistically average radius of a daughter vesicle was $R_{d}∼1.5$ μm.

**Figure 2:**
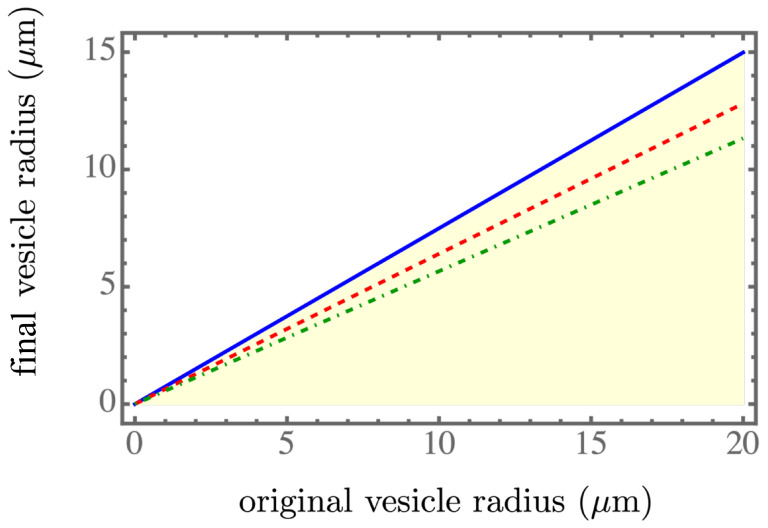
The final vesicle size $R_{1}$ as a function of the initial size $R_{0}$. The light yellow color indicates a stable regime for ΔF<0. The blue solid line, the red dashed line, and the green dash-dotted line corresponds to $\gamma_{sp}=7.05\times 10^{-6}\;\mathrm{mN}/m$, $7.01\times 10^{-6}\;\mathrm{mN}/m$, and $6.97\times 10^{-6}\;\mathrm{mN}/m$, respectively, within the spontaneous tension range in Table 1 of [7].

**Figure 3:**
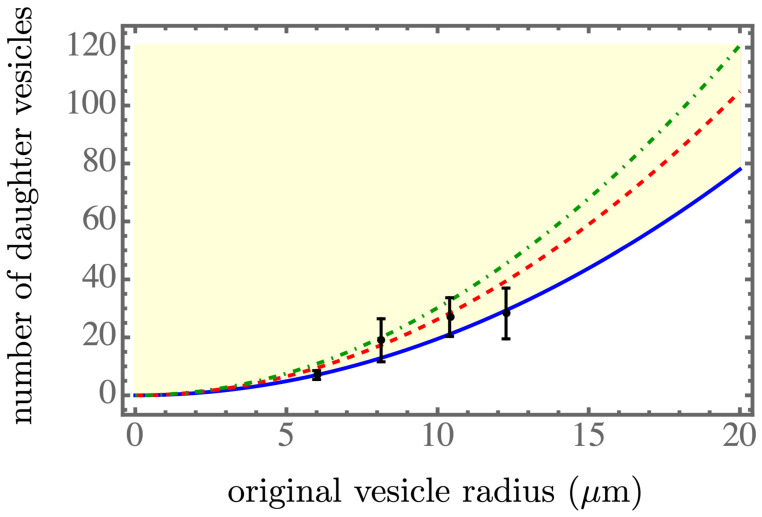
The number of daughter vesicles as a function of the initial size $R_{0}$. The black dots with the error bars are calculated from the experimental data of [3]. The light yellow color indicates the stable regime for ΔF<0. The blue solid line, the red dashed line, and the green dash-dotted line corresponds to $\gamma_{sp}=7.05\times 10^{-6}\;\mathrm{mN}/m$, $7.01\times 10^{-6}\;\mathrm{mN}/m$, and $6.97\times 10^{-6}\;\mathrm{mN}/m$, respectively, within the spontaneous tension range in Table 1 of [7].

**Figure 4:**
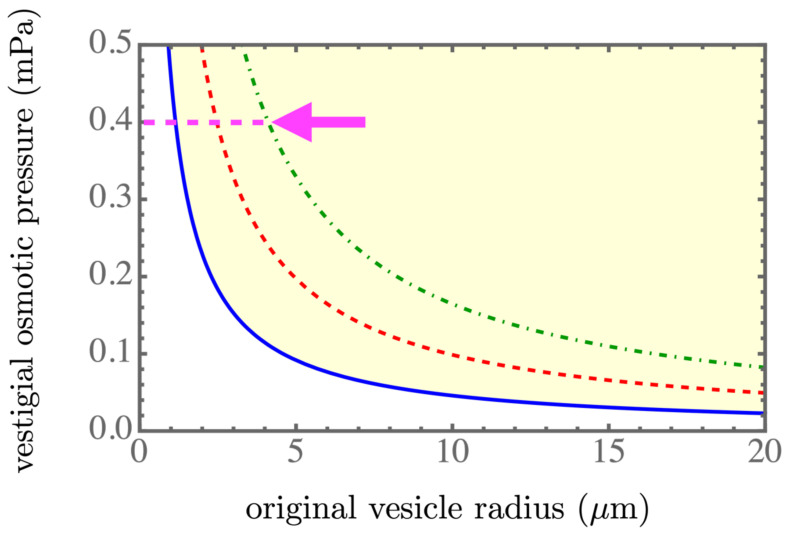
Vestigial osmotic pressure as a function of the initial size $R_{0}$. The vestigial osmotic pressure, according to the experiments in [2], was estimated to be 0.4 mPa, which is marked by a magenta arrow. The light yellow color indicates a stable regime for ΔF<0. The blue solid line, the red dashed line, and the green dash-dotted line corresponds to $\gamma_{sp}=7.05\times 10^{-6}\;\mathrm{mN}/m$, $7.01\times 10^{-6}\;\mathrm{mN}/m$, and $6.97\times 10^{-6}\;\mathrm{mN}/m$, respectively, within the spontaneous tension range in Table 1 of [7].

The equations derived for $R_{1}=R_{1}\left(R_{0}\right)$, $n=n(R_{0})$, and $\delta P=\delta P(R_{0})$ could be solved numerically, and the results are presented in Figure 2, Figure 3 and Figure 4. The light yellow color indicates a stable regime for ΔF<0. The blue solid line, the red dashed line, and the green dash-dotted line corresponds to the spontaneous tension $\gamma_{sp}=7.05\times 10^{-6}\;\mathrm{mN}/m$, $7.01\times 10^{-6}\;\mathrm{mN}/m$, and $6.97\times 10^{-6}\;\mathrm{mN}/m$, respectively, which were in the spontaneous tension range in Table 1 of [7]. We found that a smaller spontaneous tension softened the membrane of the parent vesicle to generate more daughter vesicles, as shown in Figure 3, and resulted in smaller daughter vesicles, as shown in Figure 2. More daughter vesicles, by necessity, also created more osmotic pressure, as shown in Figure 4. In the experiments, the value of the spontaneous tension was measured via the spontaneous curvature [18], which controlled membrane curvature and stabilized the multi-sphere morphologies [19].

An approximate scaling form of the solution was obtained by considering $C_{0}R_{0}\gg 1$, yielding the leading order dependence $∼1/R_{1}$ in Equation (12) and $P_{1}∼1/R_{1}$, according to $P_{1}V_{1}∼R_{1}^{2}$ in Equation (9) and similarly, $P_{0}V_{0}∼R_{0}^{2}$ in Equation (9). Considering the critical isotherm ΔF=0 in Equation (9), which was, once again, at the lowest order, we found the relation $R_{1}∼R_{0}$. However, to maintain the leading-order terms $R_{1}^{2}$ and $R_{0}^{2}$ in Equation (9), we could easily confirm the quadratic relation $n∼R_{0}^{2}$. By substituting this quadratic relation into Equation (12), we found the vestigial osmotic pressure $\delta P∼1/R_{0}$, since $n/V_{1}∼1/R_{1}$. Therefore, the behaviors of the three numerically obtained curves approximately followed linear, quadratic, and inversely linear relations, respectively.

## 4. Conclusions

The osmotic pressure plays an important role in shape change and budding [20,21]. In this study, we explored the concept of additional vestigial osmotic pressure [2] stemming from osmotically active daughter vesicles, which were fully disconnected from the lipid bilayer of the parent vesicle, and acting upon the GUV lipid membrane. This work was motivated by the recent experiments of Liu et al. [3]. After calculating the simple Helfrich elastic free energies of the initial and final states, we derived the critical isotherm that corresponded to the onset of the progeny formation inside the parent vesicle and showed that the consequences were consistent with experimental observations in [3]. While our theoretical model was simplified and could be further expanded at various levels, it did quantify the concept of the vestigial osmotic pressure and its role in the osmotic equilibrium of budding vesicles.

Our work formalized the essential concept that disconnected daughter vesicles could act as osmoticants, i.e., osmotically active solutes, and contribute additional vestigial osmotic pressure across the membrane of the parent vesicle, despite not being directly connected with other osmotically active components. While we used a simple Van’t Hoff formula for this component of osmotic pressure, it could be directly generalized, for example, to consider the interactions between the daughter vesicles confined inside the progeny vesicle, as well as to theoretically estimate osmotic pressures of various colloidal dispersions based on different models of electrostatic interactions [22].

The results presented in this work highlighted the significance of non-conventional sources of (vestigial) osmotic pressure, such as osmotically active exfoliated unilamellar vesicles [2] and osmotically active fully disconnected daughter vesicles [3]. These effects on the mechanism of osmotic regulation in cellular processes, such as fission/fusion, endocytosis, exocytosis, and nanoparticle-wrapping [23] should not be ignored.

## Data Availability

The data that support the findings of this study are available from the corresponding author upon reasonable request.
